# Exploring short- and long-term meteorological drought parameters in the Vaippar Basin of Southern India

**DOI:** 10.1038/s41598-024-62095-y

**Published:** 2024-06-11

**Authors:** Manikandan Muthiah, Saravanan Sivarajan, Nagarajan Madasamy, Anandaraj Natarajan, Raviraj Ayyavoo

**Affiliations:** 1 Agricultural Research Station, Tamil Nadu Agricultural University, Kovilpatti, Tamil Nadu India; 2 VIT School of Agricultural Innovations and Advanced Learning, Vellore Institute of Technology, Vellore, Tamil Nadu India; 3 Agricultural Engineering College and Research Institute, Tamil Nadu Agricultural University, Trichy, Tamil Nadu India; 4 Agricultural College and Research Institute, Tamil Nadu Agricultural University, Chettinadu, Tamil Nadu India; 5 Agricultural Engineering College and Research Institute, Tamil Nadu Agricultural University, Coimbatore, Tamil Nadu India

**Keywords:** Drought severity, GIS, Hazard index, Interpolation, Innovative trend, SPI, Climate sciences, Climate change, Hydrology

## Abstract

Evaluating drought parameters at the basin level is one of the fundamental processes for planning sustainable crop production. This study aimed to evaluate both short-term and long-term meteorological drought parameters within the Vaippar Basin, located in southern India, by employing the standardized precipitation index (SPI). Gridded rainfall values developed from 13 rain gauge stations were employed to calculate the SPI values. Drought parameters, encompassing occurrence, intensity, duration, frequency, and trends, were assessed for both short-term and long-term droughts. The study findings indicated that the occurrence of short-term drought was 51.7%, while that of long-term drought was 49.82%. Notably, the basin experienced extreme short-term droughts in 1980, 1998 and 2016 and long-term droughts in 1981, 2013, and 2017. Utilizing an innovative trend identification method for SPI values, a significant monotonic upwards trend was identified in October and December for short-term drought and in December for long-term drought. This study defined the minimum threshold rainfall, which represents the critical amount required to prevent short-term drought (set at 390 mm) and long-term drought (set at 635 mm). The drought severity recurrence curves developed in this study indicate that when the SPI values fall below − 1.0, short-term drought affects 25% of the basin area, while long-term drought impacts 50% of the basin area at a 20-year recurrence interval. Additionally, the drought hazard index (DHI), which combines drought intensity and severity, demonstrated higher values in the northwestern regions for short-term drought and in the southern areas for long-term drought. The study's findings, highlighting areas of drought vulnerability, severity, and recurrence patterns in the basin, direct the attention for timely intervention when drought initiates.

## Introduction

Drought is a natural disaster that inflicts the most extensive global losses and has the greatest impact among all natural disasters^1^. It is typically defined as a temporary meteorological event resulting from a prolonged absence of rainfall compared to long-term average conditions^2–4^. A deficiency in rainfall can lead to a range of consequences, affecting soil moisture, streamflow, reservoir storage, and groundwater levels. These factors, in turn, have significant repercussions on socioeconomic, agricultural, and environmental factors^5^.

Droughts develop slowly, making them difficult to detect and monitor^6^. The effectiveness of drought preparedness and mitigation efforts hinges largely on the timely acquisition of information regarding drought onset, progression, and extent. This crucial information is typically obtained through drought monitoring, which relies on drought indices. Drought indices offer decision makers insights into the severity of drought conditions and can serve as triggers for implementing drought contingency plans when available^7^. In essence, drought indices are developed based on various hydro-meteorological parameters, such as temperature, rainfall, evaporation, streamflow, and soil moisture, allowing for the assessment of different types of drought, including meteorological (related to precipitation), hydrological (related to streamflow), and agricultural (related to soil moisture) drought^8^.

Among all types of droughts, meteorological droughts are considered the most significant, as they can trigger other forms of droughts. Meteorological drought is referred as a shortage of rainfall in a specific region over a defined period^9^. Various indices have been developed to assess meteorological drought, including the Palmer modified drought index^10,11^, rainfall anomaly index^12^, standardized precipitation index (SPI)^13,14^, per cent of normal (PN)^15^, rainfall deciles^16,17^, effective drought index^18^, reconnaissance drought index^19^, and standardized precipitation evapotranspiration index (SPEI)^20^. Among these drought indices, the SPI is the most widely utilized. The World Meteorological Organization (WMO) has also endorsed the use of the SPI for drought characterization^21^.

The SPI, developed by Mckee et al.^13,14^, is employed to define, monitor, and assess drought conditions by identifying periods of insufficient rainfall across various timescales, including 1, 3, 6, 9, 12, 24, and 48 months. The National Drought Mitigation Center (NDMC) utilizes the SPI to monitor drought conditions and assess water storage conditions^22^. Numerous studies have explored the spatiotemporal variations in meteorological droughts across different regions, including Mexico^23^, Greece^24^, India^25,26^, China^27–29^, Turkey^30,31^, Iran^32,33^, the US^34^, Portugal^35^, Denmark^36^, Algeria^37^, Pakistan^38,39^, and the Kingdom of Saudi Arabia^40^. These studies assessed spatial and temporal variations in different drought severity categories using historical data, identifying the most vulnerable areas. These areas require increased attention and necessitate the implementation of effective mitigation strategies. The studies also focused on different dimensions of the drought parameters.

Indeed, the SPI has been employed in various dimensions of researching drought, covering analyses of drought occurrences, spatiotemporal assessment, frequency analysis, probabilistic characterization, trend identification, hazard evaluation, forecasting, and investigations related to climate change in India^41–43^. Moreover, many studies conducted in India in different basins have focused mainly on identifying the most vulnerable areas to different degrees of drought. For example, Patil et al. investigated the spatiotemporal characterization of droughts in terms of magnitude and severity at different return periods for various time scales in the Hyderabad Karnataka region of India^44^. Sharma et al. assessed the trends of rainfall indices and meteorological drought properties in the Mahi River basin, India^45^. The spatiotemporal drought parameters were analysed based on drought indices at different timescales over Uttar Pradesh, India, and trends were tested^46^. The spatiotemporal changes in meteorological drought were explored within the Luni River Basin in Rajasthan, India, utilizing the SPI. Drought trends were assessed through the application of the Mann‒Kendall (MK) test or the modified MK test, along with graphical innovative trend analysis (ITA)^47^.

Recently, the Innovative Trend Analysis (ITA) method, developed by Sen^48^, has garnered significant attention worldwide and has been rigorously tested and verified by numerous researchers, including Sanikhani et al.^49^ and Singh et al.^50^. One of the primary advantages of the ITA method is its capability to analyse trends and present them graphically without being constrained by factors such as nonnormality, serial correlation, or the number of data in the series^51^. Furthermore, the ITA method delivers robust and accurate results with minimal error. It also allows for the detection of both monotonic and nonmonotonic trends, categorizing time series into different subcategories, such as high, medium, and low zones^52^.

The assessment of drought hazards holds paramount significance in the context of sustainable water resource planning and management. It necessitates a comprehensive understanding of both historical drought occurrences in the region and the various concepts associated with droughts. The drought hazard index (DHI), calculated by combining occurrences of various categories of drought severities, provide an in-depth assessment of a particular region's vulnerability to drought^53^. A specific weight is assigned to each drought severity category, and the features within each category are rated to estimate the drought hazard. Integrating all severity themes allows for the creation of prepared drought hazard maps^54^. Drought hazard maps have been developed to explore the spatial attributes of drought hazards in different regions, such as Bangladesh^53^, China^54^, India^55^ and Iran^56^.

Drought is a recurring occurrence in India, predominantly impacting the Peninsular and Western regions of the country. Conversely, the central, northern, eastern, and southern parts of India experience relatively fewer instances of drought. Among India's total land area of 329 million hectares, Around 107 million hectares are experiencing different levels of water stress and drought conditions^25^. Over 100 districts spanning 13 states in India have been designated drought-prone districts, with approximately 8 of these districts located in Tamil Nadu^57^.

Drought is a natural aspect of the climate; its recurrence is an unavoidable part of our environment. Nevertheless, there is considerable confusion surrounding its characteristics. Research indicates that the inability to precisely identify drought conditions in specific scenarios has impeded our understanding of drought, resulting in uncertainty and a limited response from policymakers^3^. This study aims to investigate the short- and long-term drought parameters of the Vaippar Basin by employing the standardized precipitation index (SPI) as a drought analysis tool. The comprehensive analysis encompasses various aspects of drought, including (i) assessing drought parameters (occurrence, duration, severity), (ii) identifying historic trends, and (iii) establishing threshold rainfall values to mitigate drought, as well as (iv) developing a drought hazard index for demarcating weaker areas in the basin. This research is distinct in its approach, employing spatially interpolated gauge rainfall data at the micro-level to detect drought parameters and utilizing SPI values for innovative trend identification.

## Materials and methods

### Study area and data used

The Vaippar River Basin, situated in the southern part of Tamil Nadu, India, holds significant importance in the region's geography. It is positioned between latitudes 8° 97′ N and 9° 78′ N and longitudes 77° 24′ E and 78° 37′ E, covering a total catchment area of 5320 square kilometers. This basin is bordered by the Western Ghats to the west, the Gulf of Mannar (Bay of Bengal) to the east, the Vaigai and Gundar basins to the north, and the Tamaraparani basin to the south. It spans four districts, with Virudhunagar accounting for 68%, Thoothukudi for 20%, Madurai for 7%, and Tirunelveli for 5% of the total area.

The Vaippar River originates from the Echamalai Mottai, Neduntheri Mottai, and Kiladiparai hill ranges of the Western Ghats, near Sivagiri in the Tirunelveli district, at the uppermost elevation of 165 m above mean sea level. It flows predominantly in an eastern and southeastern direction, covering a distance of 146 km before joining the Gulf of Mannar. Within the Vaippar basin's catchment area lies hilly regions such as KodaliparaiMottai, Vasudevanallur reserve forest, and Periyasudangi Malai, among others, which receive relatively low rainfall due to their location in the rain shadow regions of the Western Ghats.

**Figure 1:**
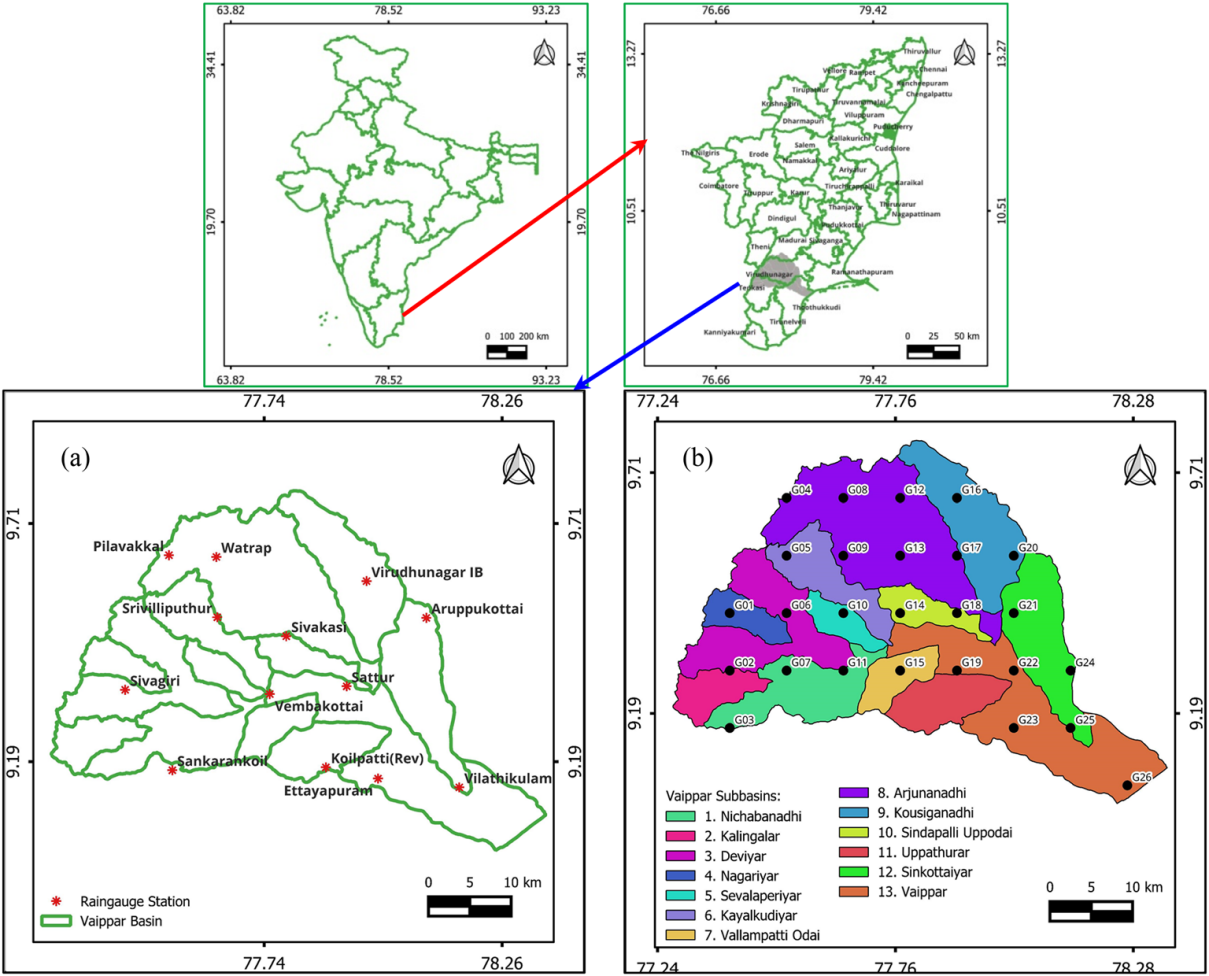
Geographical location of the study area with (**a**) raingage stations and (**b**) grid points in the subbasin.

The entire catchment area of the Vaippar Basin falls within the boundaries of Tamil Nadu State and is further subdivided into 13 subbasins, namely, Nichabanadhi, Kalingalar, Deviar, Nagariyar, Seperperiyar, Kayalkudiar, VallampattiOdai/Uppodai, Arjunanadhi, Kousiganadhi, SindapalliUppodai, Uppathurar (eastern side), Senkottaiyar, and Vaippar, as depicted in Fig. 1a,b.

This basin has a history of frequent drought occurrences, with droughts occurring every 4–8 years^58^. Approximately 74% of the basin's total geographical area is dedicated to agriculture, while forested areas account for 10%. Wasteland covers 8% of the land area, and settlements and water bodies combined occupy less than 8% of the basin's total area. Of the total agricultural land, 43% is cultivable land primarily utilized for water-intensive crops such as paddies, sugarcanes, and bananas^59^. In addition to these crops, cotton, nonpaddy crops, and dry crops are cultivated within the basin. Approximately 24% of the cultivable land is irrigated, with the remaining 76% relying heavily on rainwater for irrigation^58^.

In this study, daily rainfall data were collected from 13 rain gauge stations distributed across the Vaippar Basin. The data were sourced from the Tamil Nadu State Ground and Surface Water Resources Data Center, Public Works Department, Water Resources Organization in Chennai, Tamil Nadu, India. Table 1 provides the location details of each rain-gauge station and the period of data used in this study. Standard quality control measures were undertaken to identify outliers and errors in rainfall data for all 13 rain gauges. Any potential outliers, including missing data and instrument errors, were checked and corrected. To facilitate the analysis, the daily rainfall data for each station was processed to derive monthly data.

**Table 1:** Details of the raingage stations, geographical locations and data used for drought analysis.

Sl. no	Raingauge station	Latitude	Longitude	Elevation, m	Data availability
1	Aruppukottai	09° 30′ 09"	78° 05′ 44"	123	1971–2019
2	Virudhunagar IB	09° 35′ 00"	77° 57′ 50"	120	1971–2019
3	Sattur	09° 21′ 08"	77° 55′ 13"	91	1971–2019
4	Sivakasi	09° 27′ 44"	77° 47′ 14"	127	1973–2019
5	Srivilliputhur	09° 30′ 14"	77° 38′ 08"	146	1971–2019
6	Watrap	09° 38′ 11"	77° 38′ 01"	73	1974–2019
7	Pilavakkal	09° 38′ 25"	77° 31′ 45"	141	1982–2019
8	Koilpatti (rev)	09° 10′ 27"	77° 52′ 28"	130	1971–2019
9	Vilathikulam	09° 07′ 48"	78° 10′ 05"	47	1971–2019
10	Sankarankoil	09° 10′ 04"	77° 32′ 12"	143	1971–2019
11	Sivagiri	09° 20′ 47"	77° 26′ 01"	165	1971–2019
12	Vembakottai	09° 20′ 06"	77° 45′ 04"	111	2000–2019
13	Ettayapuram	09° 08′ 53"	77° 59′ 26"	60	1999–2019

### Methodology

**Figure 2:**
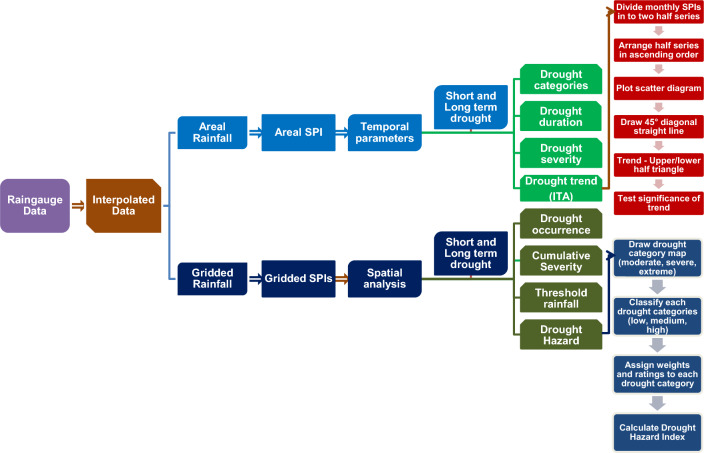
The overall process involved in drought analysis in the Vaippar Basin.

The methodology involved several steps, including the creation of gridded rainfall data by the spatial interpolation method; the calculation of basin average monthly rainfall and gridded rainfall at the time scale of 3- and 12-month; the computation of short and long-term drought data at 3- and 12-month SPI values using both average and gridded rainfall data; a temporal assessment of drought, which included the classification of SPI values into drought categories using areal SPI values; the assessment of different drought parameters; the identification of trends in the SPI series; the development of drought severity recurrence curves; the creation of maps illustrating the occurrence of drought severities; the determination of threshold rainfall to avoid drought; and the construction of drought hazard maps based on the frequency of drought events. The entire workflow is illustrated in Fig. 2.

### Spatial interpolation method for gridded rainfall data

Spatial interpolation methods play a crucial role in estimating values at sites lacking observed data utilizing known data. Several methods, including inverse distance weighting (IDW), the kriging method and splines, are commonly employed for spatial investigations of various variables^23,36,60,61^. Among these methods, the IDW approach considers the proximity of known data points to the location of interest and assigns weights accordingly, ultimately producing an interpolated surface^25,62^.

In this study, the IDW technique was employed for estimating gridded rainfall across the basin. The Thiessen polygon technique often results in a coarse approximation of rainfall spatial variation due to attribute variations associated with each rain gauge station, with variations ranging from 14 to 30%^25^. To overcome this limitation, the Vaippar Basin was subdivided into smaller square grids. Given the constraints of rainfall data availability and the uneven distribution of rain gauge stations within the basin, spatial interpolation of data at smaller grids was deemed necessary to address these challenges and achieve a more accurate representation of rainfall patterns.

The entire Vaippar basin was subdivided into 26 grids, each covering 0.125° × 0.125° and approximately 205 km^2^. These grids accounted for approximately 3.85% of the total area, equivalent to 5320 km^2^. Monthly rainfall data recorded at 13 stations were subjected to spatial interpolation using the IDW method in QGIS 3.30.2 (QGIS is an official project of the Open Source Geospatial Foundation (OSGeo) licensed under the GNU General Public License), resulting in gridded rainfall data covering the period from 1971 to 2019. Subsequently, the gridded rainfall data were used to analyse drought during both the 3- and 12-month time periods. The areal average monthly rainfall for the Vaippar Basin was computed by averaging the gridded rainfall data. The procedure employed for calculating gridded SPI values in this study is known as the 'interpolate-calculate' method, wherein rainfall is spatially interpolated first, followed by the computation of SPI values^24,25^.

### Assessing drought with the standardized precipitation index (SPI)

The standardized precipitation index (SPI), originally developed at Colorado State University in the United States, is a widely utilized tool for quantifying rainfall deficits and monitoring drought situations^13,14^. A drought event is defined as a period during which the SPI value consistently remains negative, and a drought event concludes when the SPI turns positive. The SPI can be calculated at various time periods, including 1, 3, 6, 9, 12, 24, and 48 months. Using multiple time periods enables the assessment of how a rainfall deficit impacts various water resource components, including groundwater, reservoir storage, soil moisture, and streamflow. For instance, the 3-month SPI for a specific month indicates the deviation in precipitation totals for that same month and the two preceding months, helping to gauge drought conditions.

The computation of the SPI entails modelling a probability density function to match the rainfall frequency distribution across the chosen time period. This process is conducted individually for each month (or the temporal basis of the raw rainfall time series) and for each location. Subsequently, each probability density function is standardized to a normal distribution with a mean value of zero and a standard deviation value of one. This normalization ensures that SPI values are expressed in standard deviations^62,63^. As a result, the SPI is standardized both in terms of location and time period, sharing the benefits of standardization similar to the Palmer Drought Severity Index (PDSI). Once standardized, anomaly severity was classified according to Table 2. This table also provides the associated probabilities for each severity level, derived from the normal probability density function. For instance, at a specific location in a given month, the occurrence probability for moderate droughts (SPI ≤  − 1) is 15.9%, while for extreme droughts (SPI ≤  − 2), it is 2.3%. Extreme SPI values, by definition, occur with the same frequency across all locations. A drought event is considered to commence when the SPI value consistently remains negative and concludes when the SPI becomes positive. The detailed SPI computational methodology can be found in Guttman^64,65^, McKee et al.^13,14^, and Hayes et al.^6^. The SPI has been widely employed in numerous studies to analyse meteorological droughts^24,25,33,60,66–69^.

**Table 2:** Drought classification by SPI value and corresponding probabilities.

Sl. no	Drought category		SPI	Probability (%)
1	D1	Mild drought	0 to − 0.99	34.1
2	D2	Moderate drought	− 1.00 to − 1.49	9.2
3	D3	Severe drought	− 1.50 to − 1.99	4.4
4	D4	Extreme drought	≤ − 2.00	2.3

### Temporal analysis of drought

Temporal analysis of drought was conducted in the Vaippar Basin using areal monthly SPI values and gridded SPI values. The areal monthly SPI values for short- and long-term droughts were calculated based on the areal average monthly rainfall at 3- and 12-month time periods. Following the SPI value classifications in Table 2, these values were then categorized into four groups—D1 for mild drought, D2 for moderate drought, D3 for severe drought, and D4 for extreme drought—on a monthly basis. To determine the percentage of mild to extreme drought occurrence, the proportion of months experiencing drought conditions during each SPI time period relative to the total duration of the dataset was calculated^60,62,70^.

Monthly SPI values, calculated from gridded rainfall data for each grid, were utilized to categorize drought events for each month of the year. This study also analysed the spatial evolution of severely drought-stricken areas at various severity levels over time. The primary objective was to estimate the areas covered by mild to extreme drought categories according to the frequency of drought events. To accomplish this, the number of grids expressing mild or moderate or severe or extreme drought over the specified time intervals was determined using the respective SPI values^70^. This means that the number of grids exhibiting different drought categories associated with the SPI values was determined and mapped for the period from 1971 to 2019 to observe their geographical extent.

### Analysis of drought parameters

Various drought parameters, including the most intensified drought month, annual accumulated drought severity, drought initiation and termination, drought duration, and drought severity, have been assessed for short- and long-term drought at timescales of 3- and 12-month based on run theory^71–73^. A drought event is characterized by the following parameters:

i. Most intensified drought refers to the most severe drought event during the analysis period. The deviation coefficient is determined by identifying the maximum negative departure of the SPI value from its normal value.
ii. The annual accumulated drought severity is the cumulative sum of negative SPI values throughout the year.
iii. The initiation time of a drought marks the onset of a drought event.
iv. The termination time of a drought is when the drought conditions come to an end.
v. Drought duration, stated in months, signifies the consecutive period in which a drought parameter remains below the threshold level. It spans from the initiation to the termination of a drought event.
vi. Drought severity is the aggregate deficit of a drought parameter below the threshold level, determined by summing negative SPI values during dry spells.
vii. Drought intensity, calculated as the average value of a drought parameter below the critical level, is derived by dividing drought severity by its duration.

### Developing drought severity recurrence curve

Regional drought analysis is valuable for identifying drought conditions and determining their severity within a specific year. A regional drought is considered to occur when a significant portion of the region's total area experiences drought, that is, when the cumulative area affected by local drought reaches a predefined areal threshold. Therefore, it is crucial to assess drought conditions throughout the entire region.

Solely focusing on the recurrence of drought occurrences is insufficient to gain a comprehensive understanding of droughts. It is crucial to establish quantitative relationships with other factors, such as the severity and spatial extent of droughts. This need has prompted the development of drought severity recurrence curves. These curves represent one of the most valuable methods for evaluating drought in a region. This approach was initially developed by Henriques and Santos^73^ and has been adopted by subsequent researchers, including Kim et al.^23^, Loukas and Vasiliasdes^24^, Mishra and Desai^25^, and Zhang et al.^29^. In this study, drought severity recurrence curves were developed using the average annual severity through the following methodology:

1. Estimation of the annual average severity for each grid by dividing the annual sum of negative SPI values by 12 for each time scale.
2. Derivation of drought severity with respect to spatial extents (expressed as a percentage of the basin area) considering various spatial threshold levels.
3. Evaluation of drought severity using various probability distributions to identify the best fit for recurrence interval analysis. Subsequently, conducting recurrence interval analysis employing the chosen probability distribution for each percentage of drought spatial extent to establish the connection between severity and return periods.
4. Development of line curves for average annual severity corresponding to the recurrence interval and spatial extent.

This analysis assesses annual drought severity without considering the number or duration of monthly dry periods. As a result, an extreme drought lasting for a few months may be considered equivalent in representation to a prolonged moderate dry spell. Nonetheless, this analysis offers an assessment of the degree of dryness in a given year and can be used to evaluate the impact of drought duration.

### Recurrence interval analysis of drought severity

Recurrence interval or frequency analysis is a common practice in hydrological and meteorological science and is used to assess the recurrence interval of specific events. This analysis involved the use of a selected probability distribution to estimate the recurrence interval of average annual drought severity. However, in this study, it is important to note that the annual average drought severity includes negative values. To make the model applicable for fitting into available distributions and to represent extreme conditions, these negative values of average annual drought severity were transformed into positive values. This transformation allows for the analysis of the associated risk of droughts using the exceedance probability.

Before fitting the drought severity data, various theoretical probability distributions underwent statistical testing. The most commonly used probability distributions, including normal, lognormal, gamma, and extreme value type I, were employed to determine the best-fit probability distribution for the average annual severity values of 12 month SPI values. These distributions were rigorously tested using both nonparametric Kolmogorov–Smirnov (K–S) tests and parametric chi-square tests at 5 and 1% significance levels.

The average annual drought severity (X_T_), calculated for a given recurrence interval (T), can be represented as the average values (μ) plus the deviation (ΔX_T_) of the variable from the average (X_T_ = μ + ∆X_T_). The deviation can be calculated as the product of the standard deviation (σ) and the frequency factor (K_T_), denoted as ΔX_T_ = K_T_ σ. The values of deviation (ΔX_T_) and the frequency factor (K_T_) are dependent on the chosen recurrence interval and the type of probability distribution used in the analysis^74^. The expected annual severity for various recurrence intervals, such as 2, 3, 5, 10, 20, 25, 50, 75, and 100 years, was determined using the best-fit probability distribution.

### Innovative trend identification method for short- and long-term SPI values

The innovative trend identification method has been successfully utilized for trend detection in hydrometeorological variables^48,51^. This method is simple, allowing for easy identification and visualization of trends in high, medium, and low datasets on the trend line^52^. Unlike nonparametric trend identification tests, this method does not require restrictive assumptions such as data series independence, normality, or data length^75,76^. All the data points of the time series are plotted in a Cartesian coordinate system and compared with a diagonal straight 1:1 line^49,50,77^. The computational procedure for the innovative trend method is provided below:

(a) Split the monthly SPI values into two equal halves for both the 3-month and 12-month time periods, and arrange each half series sequentially.

(b) Plot the series as a scatter diagram with the initial half of the series on the horizontal axis and the second half on the vertical axis.

(c) Draw a straight 45° line diagonally in the scatter graph representing a 1:1 relationship. Divide the plot into upper and lower half triangles.

(d) If the scattered data points align perfectly on the 45° line, the series does not exhibit a trend.

(e) When the scattered data points lie within the upper half triangle, the series is said to have an increasing trend. When the scattered points lie within the lower half of the triangle, the series is said to have a decreasing trend.

(f) A time series exhibits a monotonic trend if all scattered points are positioned either above or below the upper or lower half of the triangle. Nonmonotonic trends arise when scattered points are distributed in both the upper and lower halves of the triangle.

(g) Trends representing low, medium, and high values can be observed in the scatter graph.

(h) In assessing the significance of the trend, a null hypothesis (H0) is proposed: if the estimated slope value (S) falls below the critical threshold (S_cri_), there is no significant trend. Conversely, the alternative hypothesis (Ha) posits that a significant trend exists when S > S_cri_.

(i) The trend slope (S) is computed using the following formula^78^:

1 $$S=\frac{2({\overline{y} }_{2}-{\overline{y} }_{1})}{n}$$

where ${\overline{y} }_{1}$ and ${\overline{y} }_{2}$ represent the means of the I and II half of the SPI series, respectively, and n is the number of data points in the SPI timescales.

(j) The standard deviation (σ_s_) of the slope of the trend is calculated using the following formula^78^:

2 $${\sigma }_{S}=\frac{2\sqrt{2}}{n\sqrt{n}}\sigma \sqrt{1-{\rho }_{{\overline{y} }_{1}{\overline{y} }_{2}}}$$

where σ represents the standard deviation and ${\rho }_{{\overline{y} }_{1}{\overline{y} }_{2}}$ is the cross-correlation coefficient between the means of the two half series.

(k) The confidence limits (Scri) for a standard normal probability density function are utilized to calculate the confidence limits of the trend slope at a significance level of α, employing the following formula^78^:

3 $${CL}_{(1-\alpha )}=0\pm {S}_{cri}{\sigma }_{S}$$

If the slope falls outside the lower or upper confidence limits, the null hypothesis of no significant trend is rejected at the significance level of α. This methodology has been extensively applied to trend identification in rainfall and drought studies^50,79,80^. In this study, both short-term and long-term SPI values were employed to identify drought trends following the methodology described above.

### Threshold rainfall for no drought conditions

The SPI method offers a notable advantage by not only calculating the SPI for a specific rainfall amount but also determining threshold rainfall values corresponding to various drought severity categories at different time scales. Threshold rainfall refers to the minimal moisture necessary to establish non-drought conditions^70^. This approach introduces a new perspective to drought studies, addressing drought vulnerability by establishing specific threshold rainfall conditions.

The threshold rainfall represents the minimum amount of rainfall required to prevent drought formation. In this context, threshold rainfall values are defined as the point where the SPI value reaches zero, marking the onset of drought^70^. When the SPI value reaches zero, the threshold rainfall for a particular month equals the average monthly rainfall over the period of analysis. It is important to note that SPI values below zero signify the presence of a drought, and as these values decreases below zero, the drought severity intensifies. After calculating the threshold rainfall values for respective grid, the values are mapped to illustrate their spatial extent. This mapping aids in identifying areas where a certain amount of rainfall is required to prevent the initiation of a drought event.

### Drought hazard

The drought hazard index (DHI) was employed to assess the short- and long-term drought hazards in the Vaippar Basin over 3- and 12-month periods, aiming to facilitate mitigation plans. This index was calculated by incorporating the occurrence of moderate to extreme drought severity categories. Determination of drought severity occurrence is influenced by several key factors, including the duration of the drought period, its magnitude, and its spatial extent^70,81^. Subsequently, spatial maps based on the drought hazard index were developed to illustrate various severity levels and depict different facets of drought using GIS applications. The following methodology outlines the approach taken to create these spatial maps based on DHI values^53–56^.

i. Generate maps depicting moderate, severe, and extreme drought categories based on SPI values at 3- and 12-month intervals.
ii. Classify the maps into three categories, low, moderate, and high, with each category receiving equal representation.
iii. Subsequently, combine these maps individually to create drought hazard index maps.
iv. Assign specific weights and ratings to each drought category, as outlined in Table 6.
v. Calculate the Drought Hazard Index (DHI) for the integrated layer using the following formula: DHI = (Rating for moderate droughts × Weight assigned to moderate droughts) + (Rating for severe droughts × Weight assigned to severe droughts) + (Rating for extreme droughts × Weight assigned to extreme droughts).

### Spatial drought analysis

Spatial drought analysis was conducted using gridded SPI values to visualize the spatial extent of drought occurrence, drought severity, and threshold rainfall to avoid drought and drought hazards for short- and long-term drought at 3- and 12-month timescales within a GIS environment. The analysis was performed using QGIS 3.30.2 and involved the application of IDW techniques.

## Results and discussion

### Rainfall characteristics

The monthly rainfall data from 13 rain gauge stations were transformed into 26 gridded rainfall datasets via spatial interpolation methods within a GIS environment. The average areal monthly rainfall for the Vaippar Basin was calculated from these gridded monthly datasets, resulting in an average of 762.6 mm. Notably, 24 out of the 49 years (49%) of study recorded rainfall totals below this average.

**Figure 3:**
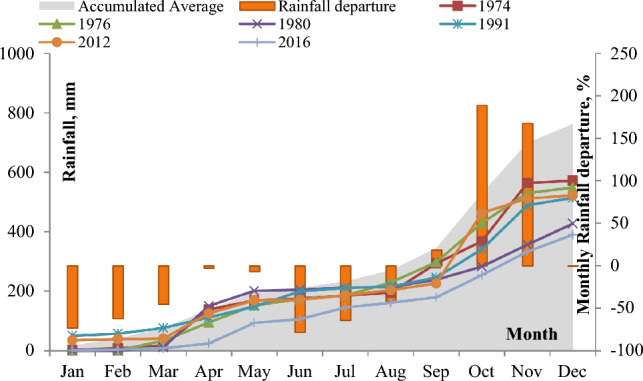
The accumulated areal monthly rainfall (mm) for selected years and the percentage of monthly rainfall departure for the Vaippar Basin.

The mean accumulated areal monthly rainfall distribution and monthly rainfall deviation from the average monthly rainfall are visually represented in Fig. 3. The maximum rainfall, reaching 183.5 mm, was recorded in October, closely followed by November, during the northeast monsoon season. In contrast, the minimum monthly rainfall of 13.8 mm was observed in June. The rainfall distribution throughout the year is as follows: 41.2 mm (5.41%) during winter, 155.7 mm (20.42%) in summer, 148.5 mm (19.48%) during the southwest monsoon, and 417.1 mm (54.69%) during the northeast monsoon. The highest rainfall occurred during the northeast monsoon season, while the rainfall during the southeast monsoon and summer monsoon seasons was approximately equal. Generally, the winter season had the lowest and least frequent rainfall. In Fig. 3, the monthly rainfall deviation from the average indicates positive values for September, October, and November, while the remaining months exhibit negative deviations.

The mean accumulated areal monthly rainfall was compared with the accumulated areal rainfall in other years throughout the study period, and the results are depicted in Fig. 3. This analysis revealed that the Vaippar Basin faced significant rainfall deficits during several decades, including the 1970s, 1980s, 1990s, and 2010s. Within these time spans, both monthly and annual rainfall often fall notably below the normal averages. Notably, in 1974, 1976, 1980, 1991, 2012, and 2016, rainfall totals were less than 75% of the average annual rainfall. Among these years, 2016, 1980, and 1991 were classified as I, II, and III extreme drought years, respectively.

**Figure 4:**
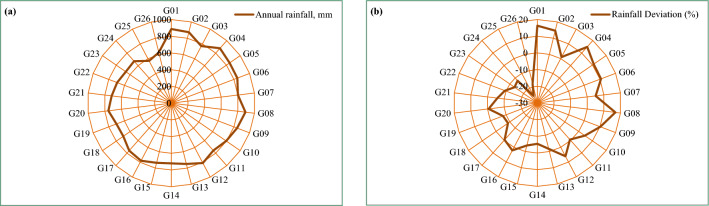
Gridwise (**a**) annual rainfall (mm) (**b**) deviation in percentage from average rainfall for the Vaippar Basin.

Figure 4a,b illustrates the annual rainfall in mm and annual rainfall deviations in percentage from the average, offering a clear visualization of both the overall variations in precipitation patterns over the study period and the gridwise distribution of these deviations. It becomes evident from the figure that the G17 to G26 grids exhibited negative values, with higher negative values corresponding to higher grid numbers. Approximately 34.6% of the rainfall grids exhibited annual rainfall exceeding 800 mm, which was predominantly concentrated in the eastern hilly region of the basin.

### Temporal drought analysis

**Figure 5:**
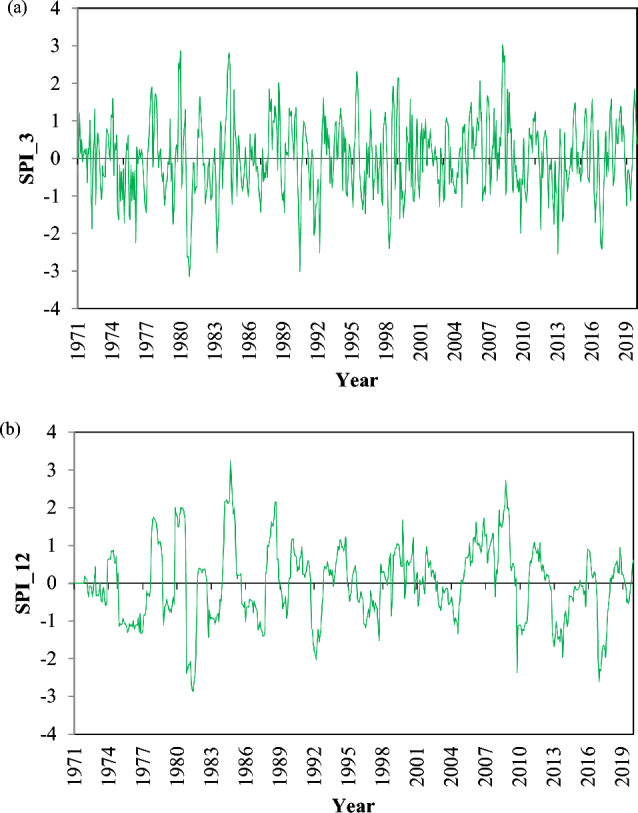
Monthly SPI values at (**a**) 3-month and (**b**) 12-month time periods for the Vaippar Basin.

The areal monthly SPI values represent drought conditions across the entire basin. These indices were computed using average areal monthly gridded rainfall data to evaluate the temporal fluctuations in drought conditions. Figures 5a,b depict the short- and long-term droughts, respectively, using the monthly SPI values at both the 3-month and 12-month time periods.

The monthly SPI values illustrate that the region experienced frequent droughts during the period under drought analysis and reveal several extreme drought events. SPI_3 values are employed to assess short-duration droughts, which occur more frequently than other droughts and impact agricultural conditions. These short-term droughts are defined as instances when the annual rainfall for three consecutive months is less than the average annual rainfall.

SPI_12 values are used to assess long-term drought. These indices are analysed to evaluate the impact of hydrological drought on water resources. The examination of long-term droughts indicates that they happen less often than short-term droughts. Long-term droughts are characterized by instances where the annual rainfall falls below the average annual rainfall.

The figures also demonstrate that drought frequency changes with varying SPI time periods. In the shorter time period (SPI_3), there was a rise in drought frequency, yet the duration of drought events decreased, leading to shorter-lasting drought events. Conversely, at longer time periods (SPI_12), droughts occur less frequently but tend to persist for more extended periods.

An examination of the estimated SPI series indicates that the basin experienced droughts of varying magnitude and durations during the drought period, with notable drought years occurring in the 1970s, 1980s, 1990s, and 2010s. Specifically, 1976, 1980, 1991, 2012, and 2016 were identified as the extreme drought years in the basin. The areal monthly SPI values at 3- and 12-month time periods were analysed, focusing on two main aspects: (i) the occurrence of drought categories expressed as percentages and (ii) the assessment of drought parameters specific to the Vaippar Basin. Furthermore, with the aid of gridded SPI values, temporal analysis was extended to assess the frequency of drought severity and its areal extent.

### Occurrence of drought severity categories

This study examined the occurrence of drought severity categories spanning from mild to extreme drought in the Vaippar Basin. This examination was carried out by calculating the proportion of each event's occurrence within its respective severity category in relation to the total number of droughts within that same severity category.

Table 3 presents the monthly distributions of occurrences of short- and long-term drought severity categories at 3- and 12-month time periods. The results suggest that mild droughts occur with the highest frequency, whereas extreme droughts are the least frequently observed for both short-term (SPI_3) and long-term (SPI_12) droughts. Furthermore, drought conditions were prevalent for almost half of the study period. The occurrence of drought severity levels, spanning from mild to extreme, demonstrates nearly similar values for both short-term and long-term droughts.

**Table 3:** Monthly distributions of the occurrence of drought severity categories expressed as percentages for the Vaippar Basin.

Month	Short-term drought (SPI_3)					Long-term drought (SPI_12)				
	D1	D2	D3	D4	Sum	D1	D2	D3	D4	Sum
Jan	36.73	12.24	0	2.04	51.01	28.57	10.20	4.08	4.08	46.93
Feb	46.94	10.20	2.04	2.04	61.22	28.57	12.24	2.04	4.08	46.93
Mar	36.73	6.12	4.08	4.08	51.01	32.65	10.20	2.04	4.08	48.97
Apr	38.78	2.04	6.12	2.04	48.98	28.57	10.20	4.08	2.04	44.89
May	34.69	10.20	8.16	0	53.05	30.61	16.33	2.04	2.04	51.02
Jun	38.78	8.16	2.04	2.04	51.02	30.61	12.24	4.08	2.04	48.97
Jul	40.82	8.16	6.12	0	55.10	26.53	14.29	4.08	2.04	46.94
Aug	26.53	14.29	4.08	2.04	46.94	32.65	16.33	0	2.04	51.02
Sep	38.78	8.16	2.04	4.08	53.06	46.94	12.24	2.04	0	61.22
Oct	30.61	4.08	4.08	4.08	42.85	46.94	6.12	2.04	2.04	57.14
Nov	42.86	6.12	0	4.08	53.06	30.61	14.29	2.04	2.04	48.98
Dec	30.61	18.37	2.04	2.04	53.06	28.57	8.16	4.08	4.08	44.89
Mean	36.91	9.01	3.40	2.38	51.70	32.65	11.9	2.72	2.55	49.82

*D1* mild, *D2* moderate, *D3* severe, *D4* extreme drought.

As shown in the table, it becomes evident that the basin faced frequent droughts throughout the year. The monthly distribution of drought occurrences during the 3-month time period indicates that negative SPI values were most frequent in February, while October recorded fewer instances of drought. On the other hand, when examining the 12-month SPI values, the occurrence of drought was greater in September, closely followed by October. Considering the significance of water resources and the predominant distribution of rainfall during the northeast monsoon, particular attention may be warranted for the month of October.

### Annual distribution of drought severity categories

**Figure 6:**
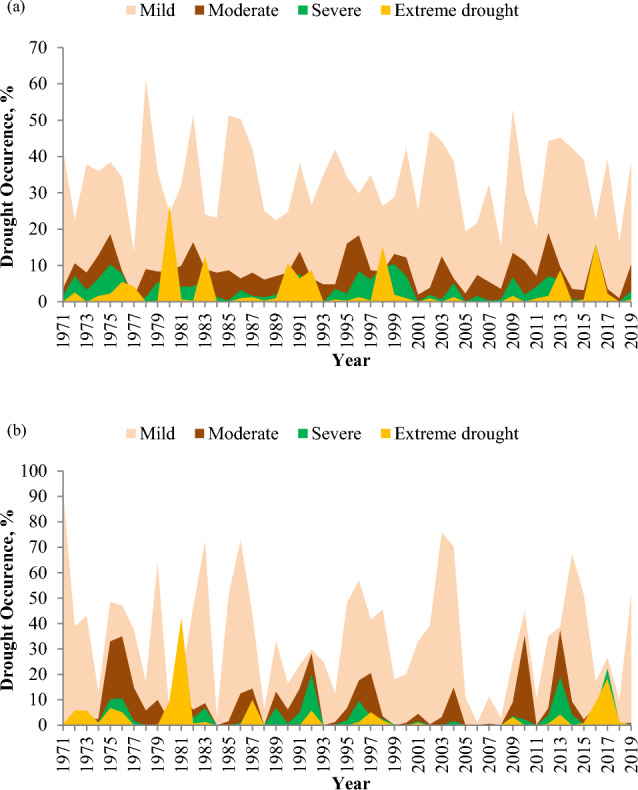
Annual distributions of drought occurrence categories (percentages) based on the (**a**) 3-month and (**b**) 12-month SPI series for the Vaippar Basin.

Monthly gridded SPI values were employed to evaluate the annual distribution of drought severity categories. For each year, for every grid, the number of months indicating mild to extreme drought conditions was used to illustrate the annual distribution of drought occurrences within different severity categories. Figure 6a,b provide visual representations of the annual distributions of drought severity categories during the study period for short- and long-term droughts at 3- and 12-month time periods, respectively.

For short-term drought, it becomes evident that in the years 1978, 1982, 2009, and 2012, more than 70% of the time experienced mild to extreme drought conditions. Conversely, the years 1975, 1980, 1985, 1986, 1991, 2000, 2013, and 2016 exhibited mild to extreme drought durations ranging from 60 to 70%. In 2016, out of the total drought occurrences, which amounted to 69.55%, approximately 47.12% of the months across all grids experienced moderate to extreme drought conditions. Likewise, in 1980 and 1998, moderate to extreme drought conditions were observed, accounting for 39.42% of the total drought occurrences out of 63.78% and 33%, respectively, of the total drought occurrences.

For long-term drought, it was observed that in the years 1971, 1975, 1976, and 2013, drought occurred more than 90% of the time. In contrast, 1981, 1983, 1986, 1992, 1996, 2004, 2010, 2014, and 2017 experienced drought rates between 80 and 90%. The most severe drought years, namely, 1981, 2013, and 2017, experienced moderate to extreme drought conditions, accounting for 66.99% of 85.58%, 60.26% of 99.04%, and 60.26% of 86.86%, respectively, of the total occurrence of drought.

### Analysis of drought parameters

The short- and long-term drought parameters derived from areal SPI values at 3- and 12-month time periods are presented in Table 4. For SPI_3, the most intensified drought, indicated by the minimum negative SPI value of − 3.14, occurred in October 1980. On the other hand, for SPI_12, the most intensified drought occurred in May and June 1981, with SPI values reaching − 2.89. The weighted annual accumulated drought severity calculated for short-term drought was greater in 1980, with a value of − 15.42, whereas for long-term drought, the severity was greater in 1981, with a value of − 21.28. This implies that during the long-term drought assessment, the severity of drought in the previous year had an impact on the following year.

**Table 4:** Drought parameters of short- and long-term SPI series for the Vaippar Basin.

Sl. no	Drought parameters	Short-term drought (SPI_3)		Long-term drought (SPI_12)	
1	Most intensified drought (year, month, SPI value)	1. Oct-1980: − 3.14 2. Jun-1990: − 3.01 3. Jan-2013: − 2.55		1. May and Jun-1981: − 2.89 2. Dec-2016: − 2.61 3. Nov-1980: − 2.39	
2	Annual accumulated drought severity	1980: − 15.42 2016: − 12.31 1998: − 8.82		1981: − 21.28 2016: − 16.65 2013: − 16.14	
3	Drought months (< 0)	302		282	
4	Drought events (nos)	91		31	
5	Average drought duration, month	3.3		9.1	
6	Most severe drought				
	Time period	Prolonged drought event duration, month	Period of prolonged drought event duration	Drought severity	Drought intensity
	SPI_3	12	07/1980–06/1981	− 17.92	− 1.49
		12	01/1982–12/1982	− 6.58	− 0.55
		9	08/1991–04/1992	− 11.27	− 1.25
	SPI_12	34	12/1974–09/1977	− 32.89	− 0.97
		31	09/2012–03/2015	− 25.79	− 0.83
		25	09/1985–09/1987	− 20.26	− 0.81

The number of drought events, drought event duration and periods of occurrence for short- and long-term drought are presented in Table 4. Drought events are defined as instances where negative SPI values persist continuously. There were 91 and 31 drought events for short- and long-term drought, respectively.

The average drought duration was computed by dividing the total number of drought months by the total number of drought events. This calculation, as shown in Table 4 for the SPI_3 and SPI_12 series, helps identify the types of droughts that occurred in the study area. Specifically, the average drought durations for short- and long-term droughts (SPI_3 and SPI_12) were 3.3 and 9.1 months, respectively, categorizing them as short- and long-term droughts, in line with the findings of Guttman^65^ and Mishra and Desai^25,62^. The study of short-term droughts is crucial for agricultural interests, while long-term droughts have significant implications for water management.

The durations of prolonged drought events, along with their severity and intensity, were identified. The prolonged drought event duration was defined as the period during which the SPI values continuously remained negative. Drought severity was determined by summing the negative SPI values for the longest duration droughts. A prolonged drought duration and the highest severity of drought were observed at SPI_12, indicating that droughts became more severe over extended periods.

Furthermore, drought intensity, calculated by dividing drought severity by the duration of drought events, was estimated. The highest intensities of − 1.49 and − 0.97 for prolonged drought events were observed for short- and long-term drought, respectively. This analysis revealed that the basin experienced its longest-duration and highest-severity droughts during the 1970s, 1980s, 1990s, and 2010s.

### Analysis of drought severity recurrence curves

Drought severity recurrence curves associated with different spatial extents serve as valuable tools for assessing both the spatial attributes and the recurrence of drought severity within a basin. These curves are particularly useful for estimating the recurrence interval associated with the average annual severity of disease in relation to its geographical extent. Furthermore, they prove instrumental in analysing the relationship between severity and recurrence interval and the spatial extent of historical droughts within the basin.

**Figure 7:**
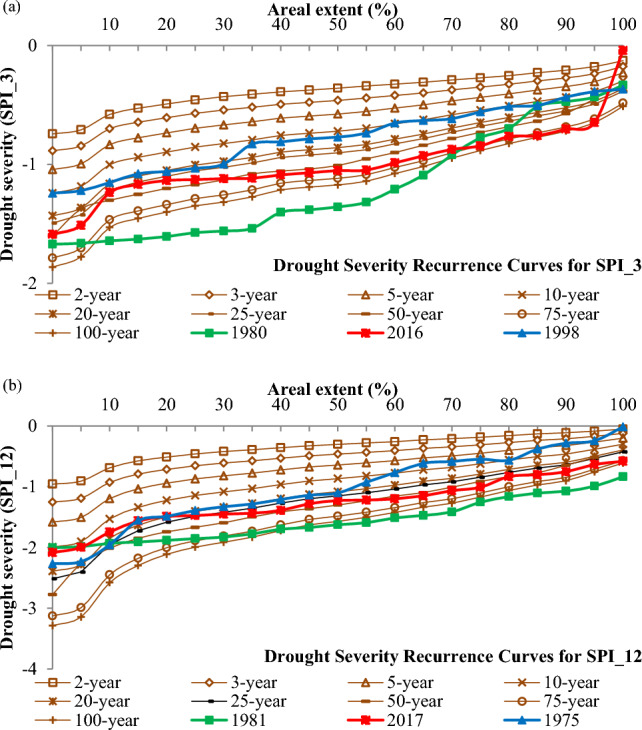
Drought severity recurrence curves (in years) of the (**a**) 3-month and (**b**) 12-month SPI series for the Vaippar Basin.

In the context of this study, drought severity recurrence curves were specifically developed based on the average annual severity for both the 3-month and 12-month time periods of SPI values. These curves are visually represented in Fig. 7, with the X-axis denoting the spatial extent of droughts and the Y-axis denoting drought severity. The average annual drought severity is calculated as the sum of negative SPI values during dry months for various return periods.

Recurrence interval analysis was performed to identify the most suitable distribution for annual drought severity. In this study, the extreme value type I distribution was chosen for recurrence interval analysis because it successfully passed both tests for the SPI_12 time period across all grids. This distribution is characterized by two parameters, and its parameter values can be estimated with relatively lower uncertainty, which is particularly important given the limited sample size in this study. Furthermore, this approach has been extensively utilized in numerous studies focused on extreme drought analysis^23–25,73^.

Figure 7a,b depict the drought severity recurrence curves for the average annual severity developed using 3-month and 12-month SPI values. The first three extreme drought years were identified based on the minimum average annual drought severity, and these years were included in the drought severity recurrence curves to identify recurrence patterns and the spatial extent of these selected drought years. Notably, most areas experienced drought in 1980, 2016, and 1998 due to short-term drought (SPI_3), with return periods of 50 years, and the spatial extent of these droughts increased over time.

The minimum average annual drought severity for long-term drought (SPI_12) occurred in 1981, 2017, and 1975. The figure illustrates that severe drought years are associated with return periods ranging from 20 to 75 years. Severe droughts (defined as SPI < − 1) exhibit lower recurrence rates and limited spatial extent.

### Analysis of drought trends

Short- and long-term drought trends of areal SPI values were evaluated using the innovative trend identification method at 3- and 12-month time periods. The trend parameters for the monthly SPI series at 3- and 12-month time periods in the Vaippar Basin, as detected by the innovative trend method, are presented in Table 5.

**Table 5:** Trend parameters detected by the innovative trend identification method for the SPI_3 and SPI_12 series of the Vippar Basin.

Series	S	ρ	σs	LCL	UCL	Decision
Short-term drought (SPI_3)						
Jan	− 0.007	0.940	0.002	± 0.003	± 0.004	− Ha**
Feb	0.001	0.971	0.001	± 0.002	± 0.003	Ho
Mar	0.007	0.949	0.002	± 0.003	± 0.004	Ha**
Apr	0.008	0.905	0.003	± 0.004	± 0.005	Ha**
May	0.009	0.949	0.002	± 0.003	± 0.004	Ha**
Jun	0.003	0.906	0.003	± 0.004	± 0.005	Ho
Jul	− 0.004	0.989	0.001	± 0.001	± 0.002	− Ha**
Aug	0.017	0.974	0.001	± 0.002	± 0.003	Ha**
Sep	− 0.004	0.939	0.002	± 0.003	± 0.004	− Ha**
Oct	0.006	0.977	0.001	± 0.002	± 0.002	Ha**
Nov	− 0.004	0.965	0.002	± 0.003	± 0.003	− Ho
Dec	0.005	0.965	0.002	± 0.003	± 0.003	Ha**
Long-term drought (SPI_12)						
Jan	0.002	0.961	0.002	± 0.003	± 0.003	Ho
Feb	0.001	0.982	0.001	± 0.002	± 0.002	Ho
Mar	0.002	0.98	0.001	± 0.002	± 0.002	Ho
Apr	0.002	0.962	0.002	± 0.003	± 0.003	Ho
May	0.002	0.952	0.002	± 0.003	± 0.004	Ho
Jun	0.002	0.974	0.001	± 0.002	± 0.003	Ho
Jul	0.001	0.952	0.002	± 0.003	± 0.004	Ho
Aug	0.002	0.952	0.002	± 0.003	± 0.004	Ho
Sep	0.003	0.941	0.002	± 0.003	± 0.004	Ho
Oct	0.001	0.932	0.002	± 0.004	± 0.004	Ho
Nov	0.001	0.97	0.001	± 0.002	± 0.003	Ho
Dec	0.003	0.961	0.002	± 0.003	± 0.003	Ha**

*S* slope, *ρ* correlation coefficient between the means of two half series, *σs* standard deviation of trend slope, *LCL/UCL* lower/upper confidence limits, *Ho* no significant trend, *Ha* significant trend.

**Significance at 5 and 10%, respectively.

Based on the observations from the table, it is evident that the slope values of several monthly SPI series during the period 1971–2019 fall outside the lower and upper confidence limits (CL), indicating the presence of a significant trend in the rainfall pattern. Furthermore, there is a notable correlation between the two halves of the SPI series. Specifically, a significant upwards trend was identified in the months of March, April, May, August, October, and December, while a significant downwards trend was observed in January, June, and November, both at the 5% and 10% significance levels for short-term drought. Additionally, a significant upwards trend was noted in December at both the 5% and 10% significance levels for long-term drought.

**Figure 8:**
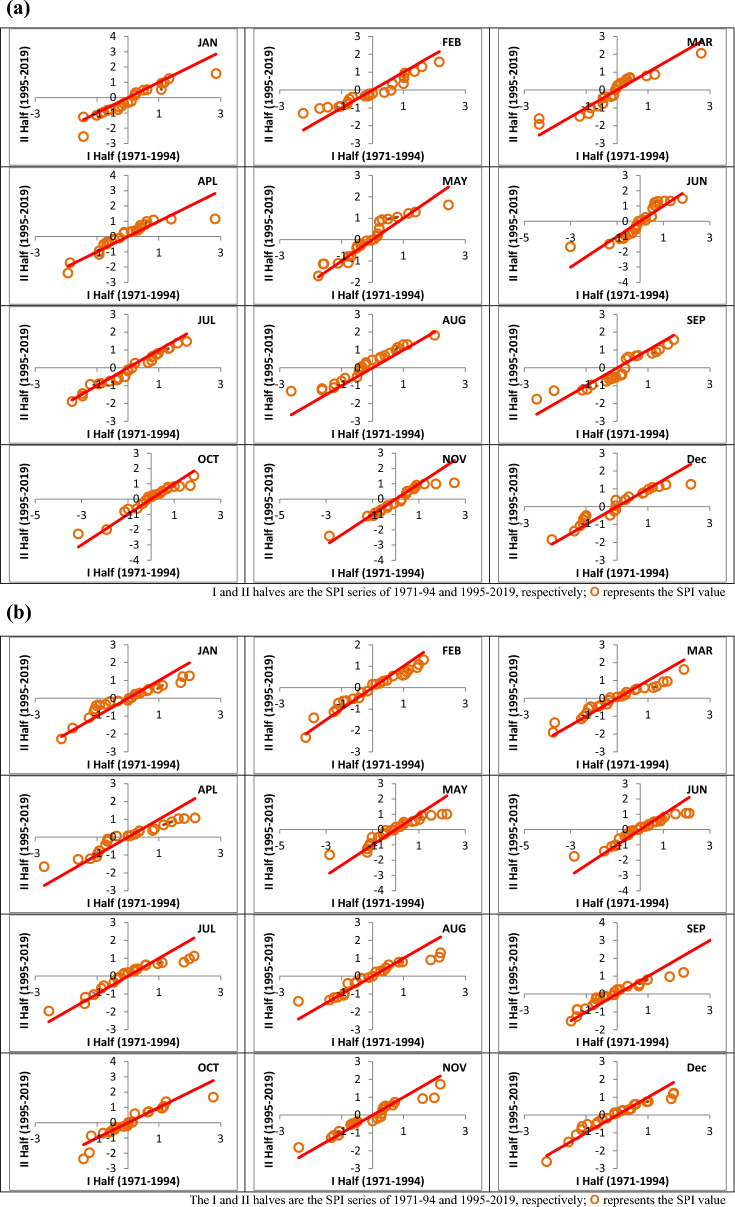
(**a**) Short-term drought trend determined by the innovative trend identification method for the Vaippar Basin. (**b**) Long-term drought trend determined by the innovative trend identification method for the Vaippar Basin. I and II halves are the SPI series of 1971–94 and 1995–2019, respectively; orange circle represents the SPI value.

Figure 8a,b present a visual representation of the scattered SPI values of different months for short- and long-term droughts. The entire SPI series was split into two equal halves, with the first half plotted against the second half. One of the key advantages of this method is its ability to discern subtrends within an SPI series^48^. By examining the distribution of the data points on the ITA graph, the time series were categorized into three zones, namely, low, middle, and high, along the 1:1 line, facilitating detailed interpretation. Furthermore, the scatter plot technique allows for the identification of both monotonic and nonmonotonic upwards or downwards trends within a series by examining the positions of data points. A data point that aligns along the 1:1 line indicates the absence of a trend. However, if data points are situated in either the upper or lower triangle regions, this indicates a monotonic trend. On the other hand, if some data points cross the 1:1 line, it is considered a nonmonotonic trend. From the figures, it becomes evident that the majority of SPI points are situated in the upper triangle and are predominantly concentrated on the inner side of the graph. This pattern indicates a monotonic upwards trend in the data for most months.

### Spatial drought analysis

Although estimating drought parameters at an areal scale offers valuable insights for water management, it is imperative to assess drought within a basin on a location-specific basis. Spatial drought analysis is essential for comprehending the spatial distribution of drought parameters and pinpointing the regions most impacted during a specific drought event.

In this study, spatial drought analysis was conducted using gridded SPI values estimated for short- and long-term droughts using the SPI_3 and SPI_12 time periods. The spatial analysis was carried out using QGIS 3.30.2, employing the IDW method to visualize the distribution of various drought parameters across the Vaippar Basin. The Vaippar Basin was divided into distinct regions: (a) the western side (Kalingalar, Deviar, and Nagariyar); (b) the central part (Sevalperiyar, Kayalkudiar, and SindapalliUppodai); (c) the southern side (Nichabanadhi, VallampattiOdai/Uppodai, and Uppathurar); (d) the northern side (Arjunanadhi and Kousiganadhi); and (e) the eastern side (Senkottaiyar and Vaippar).

Various types of maps were created to visualize the spatial distribution of drought parameters, including (i) occurrence of drought categories as a percentage, ranging from moderate to extreme severity (SPI < − 1); (ii) weighted annual accumulated severity and identification of the worst drought years; (iii) threshold rainfall values required to avoid drought; and (iv) the drought hazard index. These maps help provide a comprehensive understanding of the spatial dynamics of drought conditions within the Vaippar Basin.

### Spatial analysis of occurrence of drought severity categories

**Figure 9:**
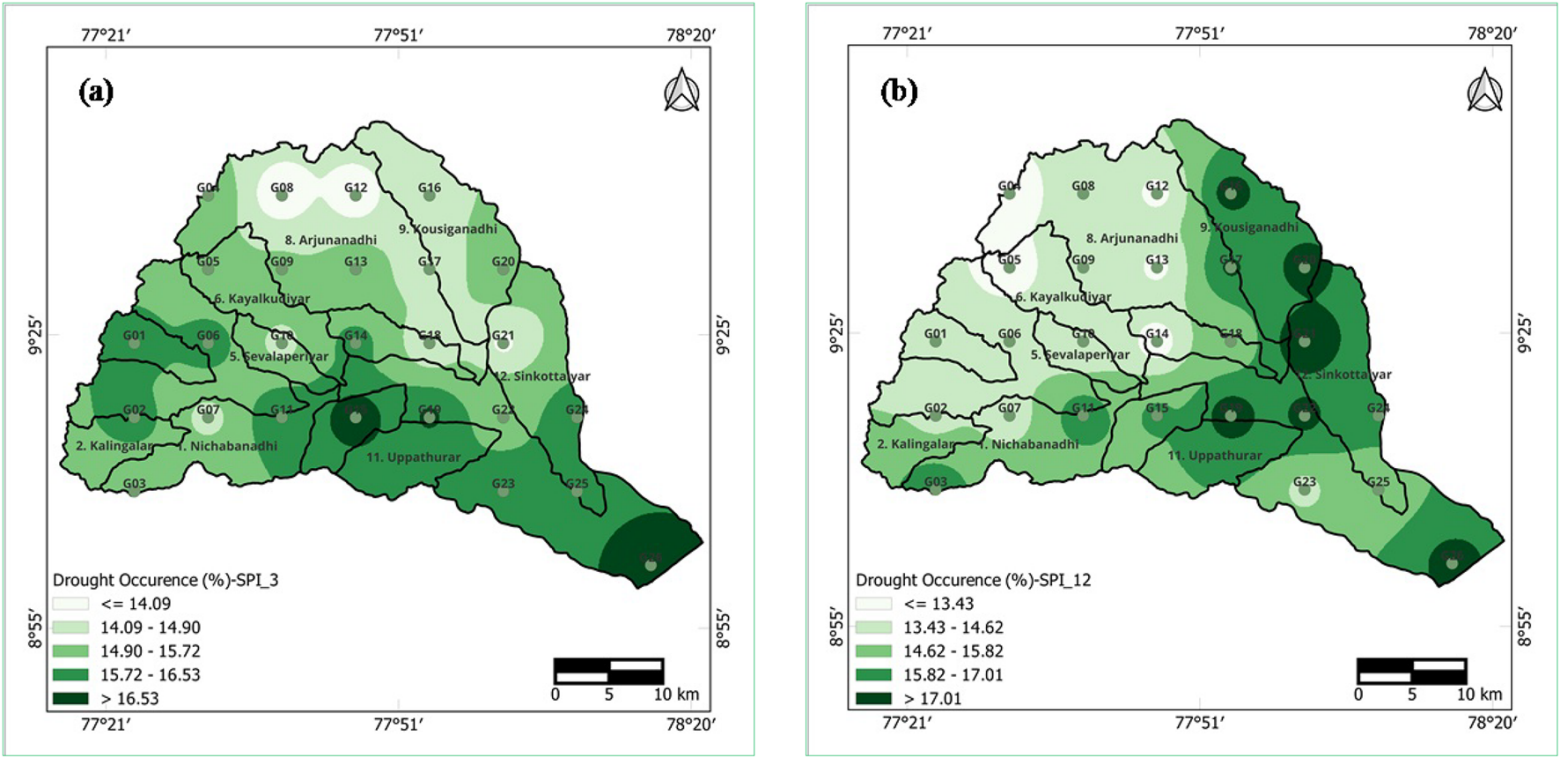
Occurrence of drought for (**a**) 3-month and (**b**) 12-month SPI series in the Vaippar Basin.

Analysing the percentage occurrence of drought across different severity categories within the Vaippar Basin offers valuable insights for identifying regions that experience recurring droughts. The spatial analysis of the occurrence of drought severity categories as a percentage based on SPI values at 3- and 12-month time periods is depicted in Fig. 9a,b. To create these maps, the percentage of severe drought occurrences was calculated by considering instances of moderate, severe, and extreme drought (SPI > − 1) categorizations.

In the case of short-term drought in the 3-month SPI series, drought was less prevalent on the northern side (Arjunanadhi and Kousiganadhi) than on the eastern side (Senkottaiyar and Vaippar). On the other hand, when examining long-term drought in the 12-month SPI series, drought occurrence was found to increase progressively from the western side (Kalingalar, Deviar, and Nagariyar) toward the eastern side (Senkottaiyar and Vaippar).

It has been observed that there is a distinct relationship between short-term and long-term droughts in most places, except for a few exceptions. In the southern mountainous region of the study area, rainfall typically exceeds normal levels compared to those in the central and northern parts of the basin. However, despite receiving more rainfall, these southern areas are more frequently affected by drought. This has significant implications for water resources, as it leads to a reduced water supply to the lower parts of the basin.

### Spatial analysis of weighted annual accumulated drought severity

The weighted annual accumulated drought severity for every grid was computed by multiplying the accumulated severity (sum of all negative values) during monthly dry spells by the probability of occurrence of drought for each year and each grid. The probability of occurrence of drought for each year and each grid was estimated by dividing the number of drought months (i.e., months with negative SPI values) by twelve^23–25,29^. In this analysis, each instance of drought can be consistently linked to a particular year, preventing intermittence and implicitly accounting for the duration of dry spells within that specific year.

**Figure 10:**
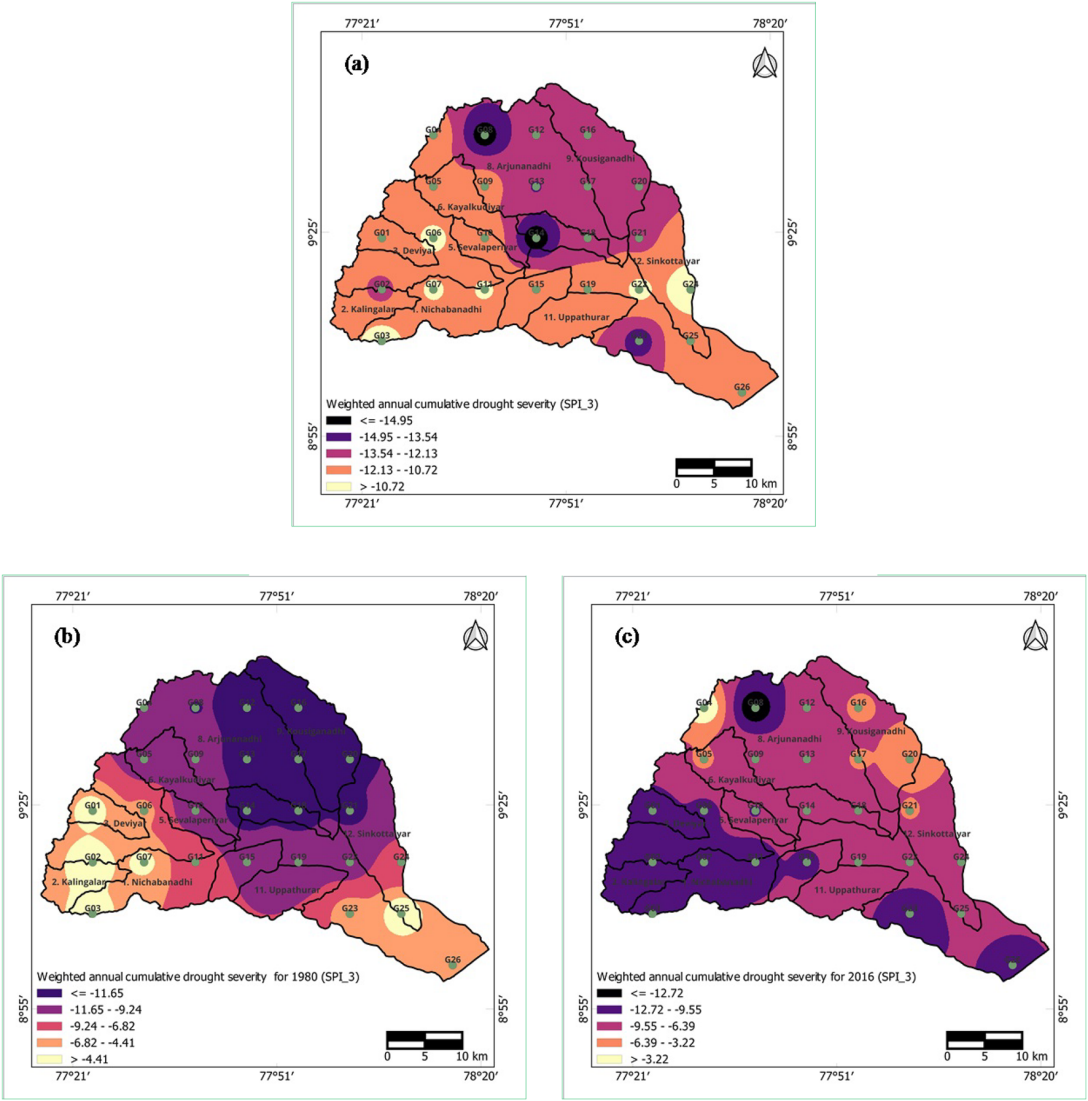
Weighted annual accumulated drought severity of short-term drought at 3-month time period for (**a**) annual minimum severity, (**b**) severity during 1980, and (**c**) severity during 2016 for the Vaippar Basin.

The spatial distribution of the worst drought years, as determined by the estimated weighted annual accumulated drought severity during the 3-month time period, is illustrated in Fig. 10a–c. The worst drought year was selected based on the year with the lowest accumulated severity values. In particular, 1980 and 2016 emerged as the worst drought years during the 3-month time period. Drought severity exhibited higher values in the northern regions (Arjunanadhi and Kousiganadhi) as well as in the central region (SindapalliUppodai) of the basin. For the 1980 drought, severe drought was predominantly observed in the northern areas, while for the year 2016, drought severity was notably greater in the southwestern regions (Nichabanadhi, Kalingalar, Deviar, and Nagariyar) as well as in certain parts of the Vaippar and Arjunanadhi subbasins.

**Figure 11:**
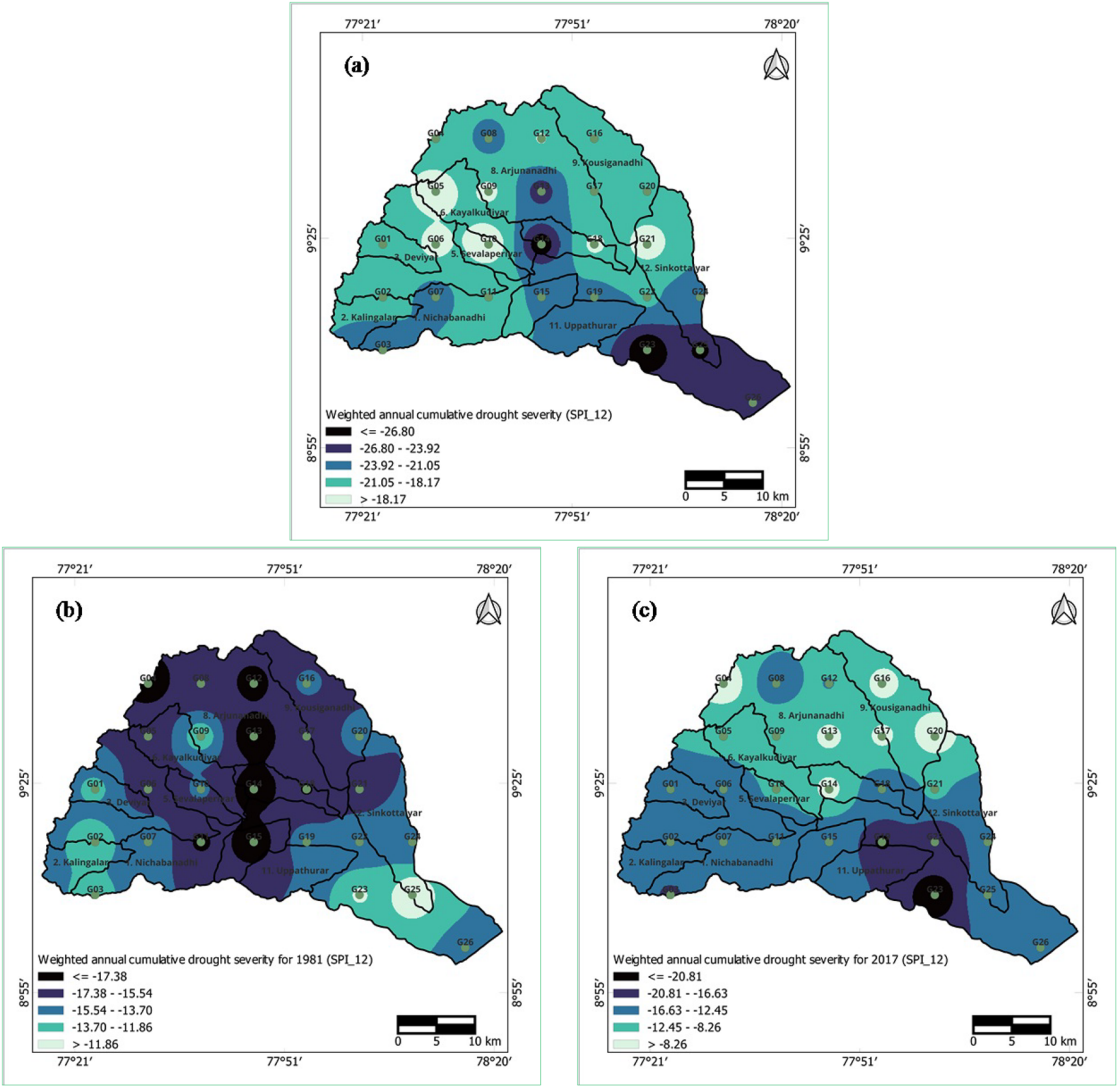
Weighted annual accumulated drought severity of long-term drought at a 12-month time period for (**a**) annual minimum severity, (**b**) severity during 1981, and (**c**) severity during 2017 for the Vaippar Basin.

The spatial distributions of the extreme drought years and weighted annual accumulated drought severity during the 12-month time period are illustrated in Fig. 11a–c. At the 12-month SPI time period, the years 1981 and 2017 exhibited heightened severity, attributed to the effects of the 1980 and 2016 droughts, along with accumulated rainfall from the preceding year. Weighted annual accumulated severity scores were notably greater for SPI_12 than for the SPI_3 series. For 1981, drought severity was most pronounced in the central and northern regions of the basin, while in the case of 2017, greater drought severity was observed in the lower regions of the basin.

### Spatial analysis of threshold rainfall for normal/nondrought conditions

**Figure 12:**
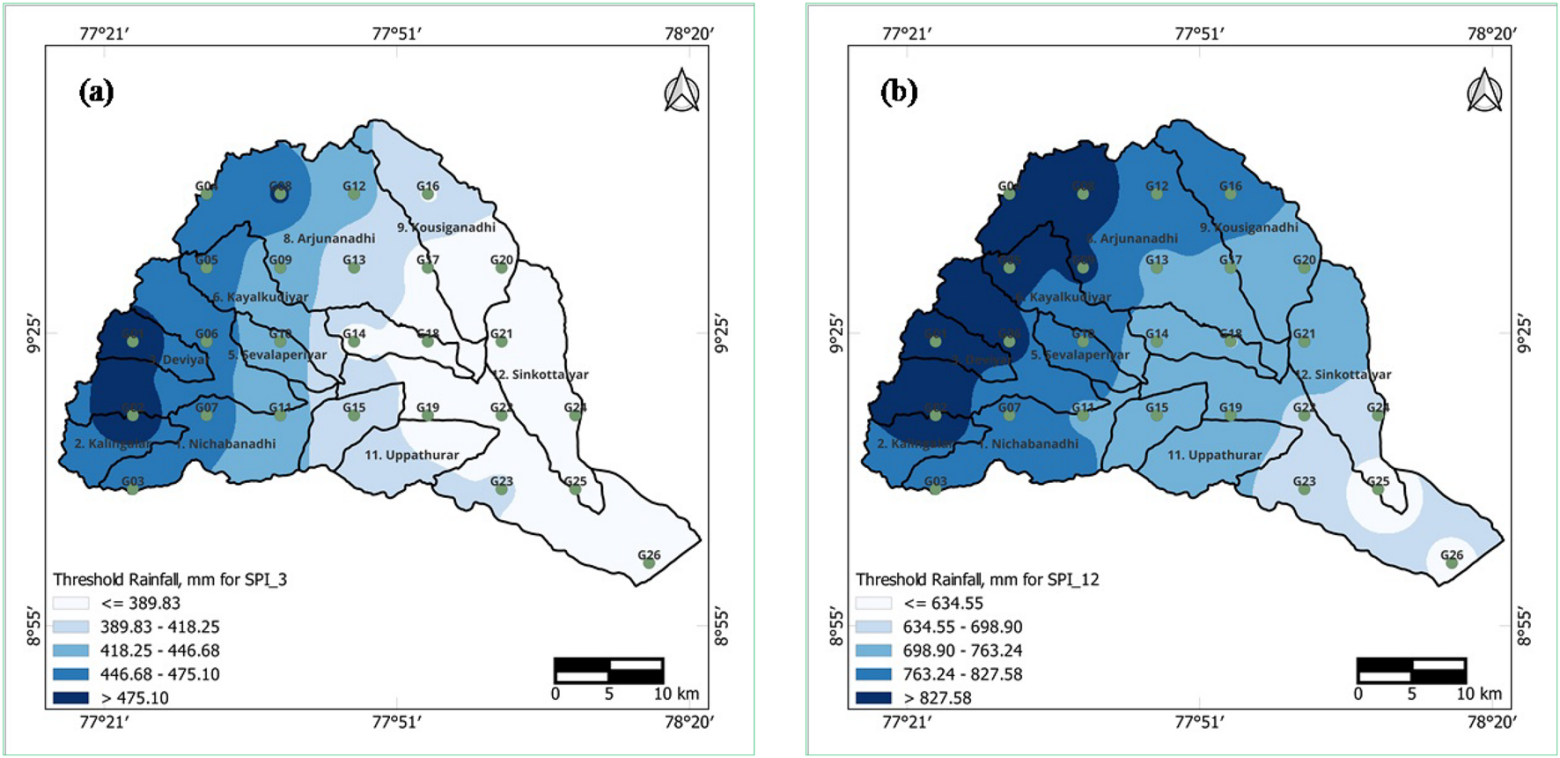
Threshold rainfall (mm) for (**a**) 3-month and (**b**) 12-month SPI series of the Vaippar Basin.

The spatial analysis of the threshold rainfall for normal/nondrought conditions for short- and long-term drought events at 3- and 12-month time periods is presented in Fig. 12a,b. By referring to these figures, it can be observed that the rainfall demand for normal/nondrought occurrences increases from east to west in the Vaippar Basin. Short-term drought occurred during the 3-month time period in December, which covers the northeast monsoon season from October to December over the Vaippar Basin, and the threshold rainfall values for normal conditions vary from 390 to 475 mm. Rainfall values increase from the eastern parts toward the western parts and reach their maximum in the southwestern parts of the basin.

The threshold rainfall values for long-term drought during the 12-month time period exhibit spatial patterns resembling those observed in short-term drought during the 3-month time period, with threshold rainfall ranging from 635 to 828 mm. It is evident that the geographical distribution of these threshold values displays moderate differences. For instance, approximately 828 mm of rainfall is necessary to avert drought in the western regions (Deviar, Nagariar, Kousiganadhi, and Arjunanadhi subbasins), whereas the rainfall requirement for normal/nondrought conditions decreases to 635 mm in the eastern regions (Vaippar subbasin).

Notably, the Deviar, Nagariar, Kousiganadhi, and Arjunanadhi subbasins, located in the hilly region of the Vaippar Basin, usually receive more rainfall than other regions. These areas are likely to be more susceptible to long-term droughts. Areas that typically receive more rainfall are more severely affected by drought events than are other areas that receive less rainfall.

### Spatial analysis of drought hazard

**Figure 13:**
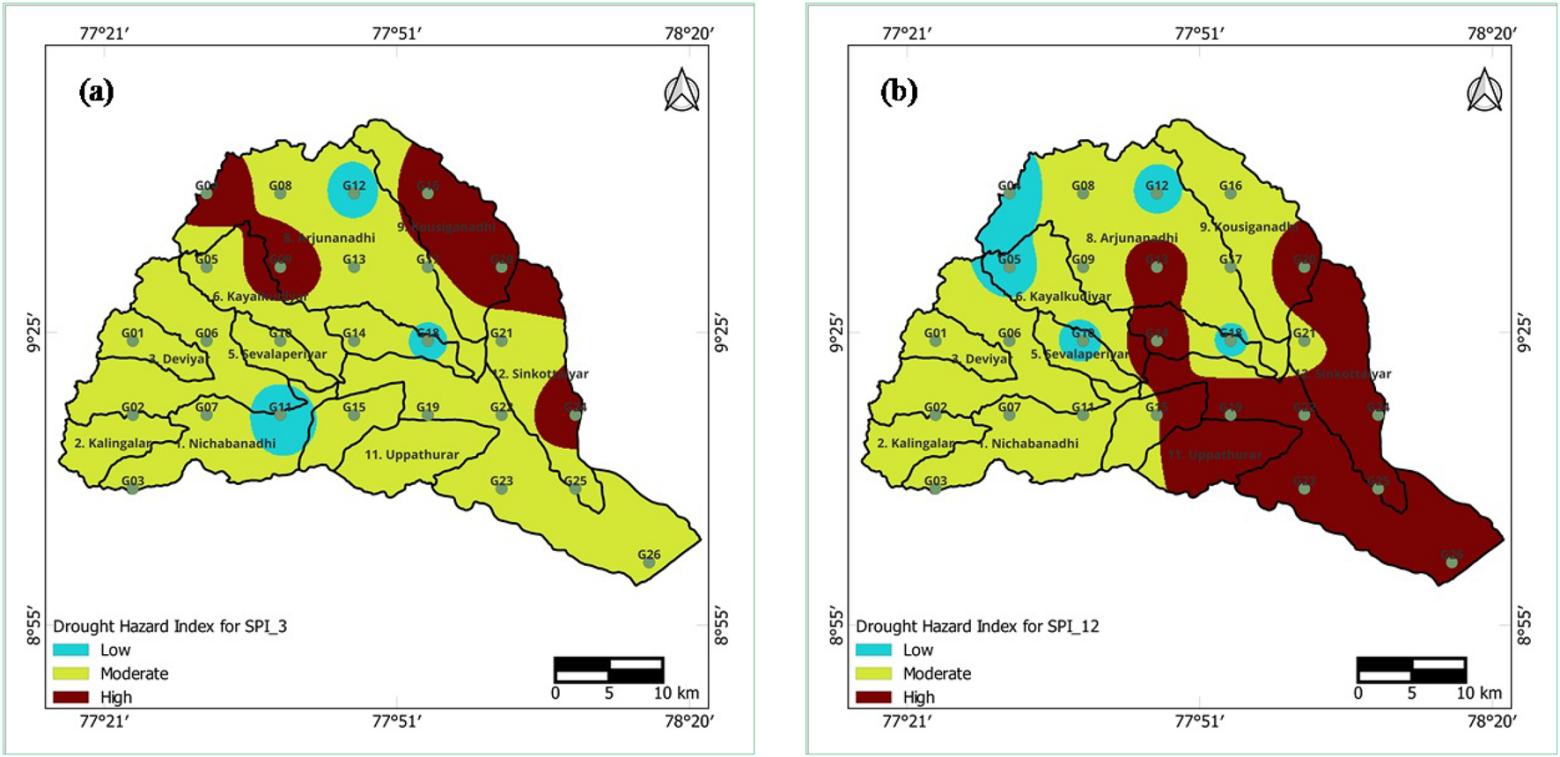
Drought hazard indices for the (**a**) 3-month and (**b**) 12-month SPI series of the Vaippar Basin.

The drought hazard index (DHI) was calculated by integrating the incidences of moderate, severe, and extreme drought categories for short- and long-term droughts at 3- and 12-month time periods. The integrated layers were then superimposed on the subbasin map of the Vaippar Basin. DHI values are categorized into three classes using the ratio 33.3:33.3:33.4 for both the 3- and 12-month time periods, as illustrated in Table 6. The spatial extent of the DHI is presented in Fig. 13a,b.

**Table 6:** Weights and ratings assigned to occurrence of drought categories and classification and area covered by the drought hazard index.

Level of severity	Ratings	Occurrence of drought severity categories (%)			DHI	Area covered, %
		Moderate drought	Severe drought	Extreme drought		
		Weight = 1	Weight = 2	Weight = 3		
Short-term drought (SPI_3)						
Low	1	5.95–7.76	2.38–3.40	1.70–2.49	8–10	19.23
Moderate	2	7.77–9.58	3.41–4.42	2.50–3.29	11–12	61.54
High	3	9.59–11.39	4.43–5.44	3.30–4.08	13–15	19.23
Long-term drought (SPI_12)						
Low	1	5.44–7.71	0.85–3.0	1.53–2.95	6–8	30.77
Moderate	2	7.72–9.97	3.01–5.16	2.96–4.36	9–10	42.31
High	3	9.98–12.24	5.17–7.31	4.37–5.78	11–13	26.92

For short-term droughts at 3 months, high DHI values covered 19.23% of the total land area, primarily in minor parts of the northeastern and northwestern areas (Arjunanadhi and Kousiganadhi) of the basin. Moderate hazards covered 61.54% of the total land area and were predominant in most parts of the basin. Low-hazard areas covered 19.23% of the total land area and were found mainly in very small sections.

After long-term drought for 12 months, higher DHI values were distributed across 26.92% of the total land area, largely in the lower parts of the basin (Uppathurar, Senkottaiyar, and Vaippar). Moderate drought hazards covered 42.31% of the total area and were distributed in the western and central regions. Low drought hazards covered 30.77% of the area and were primarily located in the western parts.

Assessing the spatial and temporal variability of short- and long-term meteorological drought parameters is the first step in managing drought-related risk^82^. This study revealed that droughts are recurrent phenomena in the Vaippar Basin. A decrease in the percentage of actual rainfall from normal rainfall in the rainy season has led to more severe and longer-lasting droughts. The present study revealed that for short-term drought, 1978, 1982, 2009, and 2012 had mild to extreme drought conditions for more than 70% of the time. For long-term droughts, drought occurred more than 90% of the time in 1971, 1975, 1976, and 2013. The weighted annual accumulated drought severity was greater in 1980 for SPI_3, with a value of − 15.42, whereas for SPI_12, the severity peaked in 1981, with a value of − 21.28. Notably, the highest intensity, at − 1.49, was observed in SPI_3 in comparison to SPI_12, which had an intensity of − 0.97. Most parts of the basin experienced drought in 1980, 2016, and 1998 due to short-term drought, with return periods of 50 years. Long-term droughts occurred in 1981, 2017, and 1975, with return periods ranging from 20 to 75 years. This study revealed a significant upwards monotonic trend in the monthly SPI series for both short- and long-term droughts utilizing an innovative trend identification method.

The spatial analysis revealed that the occurrence of drought was greater on the eastern side (Senkottaiyar and Vaippar) for both short- and long-term droughts. Drought severity exhibited higher values in the northern regions (Arjunanadhi and Kousiganadhi) for short-term drought, whereas severity scores were notably greater in the eastern parts of the basin for long-term drought. Threshold rainfall values to avoid drought increased from the eastern parts toward the western parts, reaching their maximum in the southwestern parts of the basin for both short- and long-term drought. Furthermore, the drought hazard index was greater in northwestern areas (Arjunanadhi and Kousiganadhi) for short-term drought and in southern parts (Uppathurar, Senkottaiyar, and Vaippar) for long-term drought.

The occurrence of drought in highly elevated eastern parts of the basin reduces the inflows to the lower parts of the basin and has a great impact on both rainfed and irrigated agriculture. On the other hand, increasing the occurrence of drought in the western parts of the basin, particularly in the rainfed areas of the basin, will further complicate agricultural-dependent farmers’ livelihoods. The increasing occurrence of meteorological droughts coupled with hydrological droughts in the upper Vaippar basin will have significant implications for the water supply in the lower basin. Access to site-specific data regarding drought occurrence, severity, threshold rainfall, and the drought hazard index could prove invaluable for decision-makers. This information can assist in identifying suitable mitigation measures for future drought events and in mitigating their adverse effects.

Studies of a similar nature, investigating rainfall pattern rainfall deficiency, have been previously conducted in India^45–47,83^. In this regard, drought analysis studies can provide valuable information for forecasting and mitigating drought impacts at the basin scale^23,84–86^. Studies also support the effectiveness of innovative trend analysis in detecting trends^46^. The drought vulnerability map developed in this study will be highly useful in identifying vulnerable areas within the study area for various degrees of drought, a finding also corroborated by previous research^56^. While our study assessed drought parameters using historical data, early warning systems that utilize real-time, high-resolution satellite rainfall products present a superior option for timely information on drought onset during various growing seasons^87^.

## Conclusions

This study aimed to assess short- and long-term drought parameters in the Vaippar Basin using gridded rainfall data and the standardized precipitation index (SPI) at 3- and 12-month time periods. Temporal analysis included evaluating drought parameters such as the occurrence of drought severity categories, drought events, duration of drought event, severity, drought trend and drought severity recurrence curves. Spatial analysis utilizing gridded SPI values for short- and long-term drought encompassed the occurrence of drought categories, weighted annual accumulated severity, threshold rainfall values, and drought hazard indices. The results of the study showed that mild droughts were the most common droughts, while extreme droughts were the least common for both short-term and long-term droughts. Additionally, nearly half of the study period experienced drought conditions. The study showed that short-term drought events had an average duration of 3.3 months, while long-term droughts lasted for an average of 9.1 months. This study revealed a significant upwards monotonic trend in the monthly SPI series for both short- and long-term droughts utilizing an innovative trend identification method. The spatial analysis revealed that the occurrence of drought was greater on the eastern side for both short- and long-term droughts. Furthermore, the drought hazard index developed based on drought severity was greater in northwestern areas for short-term drought and in southern parts for long-term drought. Data pertaining to location-specific drought hazards, including information on drought occurrence, severity, threshold rainfall, and the drought hazard index, could prove instrumental for decision makers. Such information can aid in the identification of suitable mitigation strategies for future drought events and in mitigating their potential impacts. The core strength of this study lies in integrating all drought parameters to create a micro-level drought vulnerability map in the study area, alongside assessing trends. Advancing, the subsequent stage of this research will involve predicting drought trends, thus enabling better planning for the future within the basin. Additional research is necessary to gain a deeper understanding of the effects of droughts on water resources, encompassing streamflows and groundwater, and water storage structures throughout the basin.

## Data Availability

The data used to support the findings of this study are available from the corresponding author upon request.
